# GenVisR: Genomic Visualizations in R

**DOI:** 10.1093/bioinformatics/btw325

**Published:** 2016-06-10

**Authors:** Zachary L. Skidmore, Alex H. Wagner, Robert Lesurf, Katie M. Campbell, Jason Kunisaki, Obi L. Griffith, Malachi Griffith

**Affiliations:** ^1^McDonnell Genome Institute, Washington University School of Medicine, St. Louis, MO 63108, USA; ^2^Department of Medicine; ^3^Siteman Cancer Center; ^4^Department of Genetics, Washington University School of Medicine, St. Louis, MO 63110, USA

## Abstract

**Summary:** Visualizing and summarizing data from genomic studies continues to be a challenge. Here, we introduce the GenVisR package to addresses this challenge by providing highly customizable, publication-quality graphics focused on cohort level genome analyses. GenVisR provides a rapid and easy-to-use suite of genomic visualization tools, while maintaining a high degree of flexibility by leveraging the abilities of ggplot2 and Bioconductor.

**Availability and Implementation:** GenVisR is an R package available via Bioconductor (https://bioconductor.org/packages/GenVisR) under GPLv3. Support is available via GitHub (https://github.com/griffithlab/GenVisR/issues) and the Bioconductor support website.

**Contacts:** obigriffith@wustl.edu or mgriffit@wustl.edu

**Supplementary information:** Supplementary data are available at *Bioinformatics* online.

## 1 Introduction

The continued development of massively parallel sequencing technologies has led to an exponential growth in the amount of genomic data produced (Kodama *et al.*, 2012). This growth has in turn enabled scientists to investigate increasingly large, cohort-level genomic datasets. Generating intuitive visualizations is a critical component in recognizing patterns and investigating underlying biological properties in cohorts under study. A significant bottleneck exists, however, between data generation and subsequent visualization and interpretation (Good *et al.*, 2014). Additionally, generating publication-quality figures for effective communication of these data typically requires *ad hoc* methods such as manual creation or extensive graphic manipulation with third party software. This process is both time intensive and difficult to automate/reproduce. Further, the absence of software supporting multiple species can make this process even more demanding. Here, we present GenVisR, a Bioconductor package to address these issues. GenVisR provides a user-friendly, flexible and comprehensive suite of tools for visualizing complex genomic data in three categories (small variants, copy number alterations and data quality) for multiple species of interest.

## 2 Visualization of small variants

**Fig. 1.**
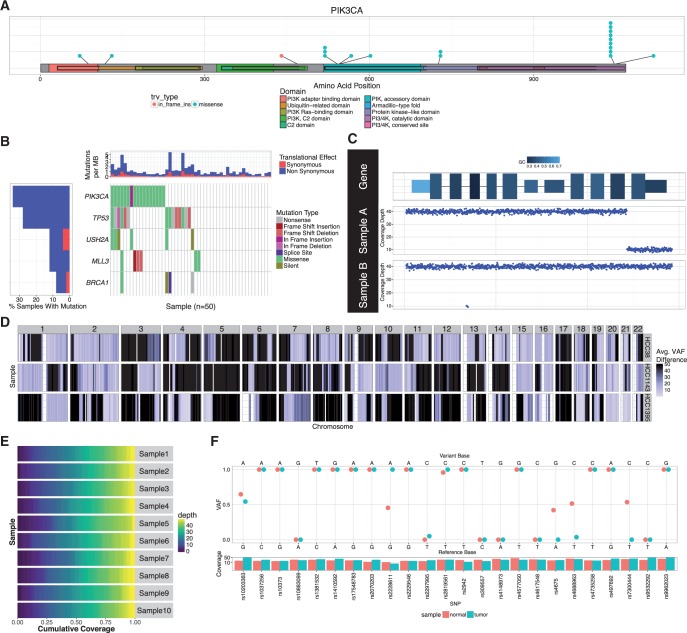
Selected representation of GenVisR visualizations. (**A**) Output from lolliplot for select TCGA breast cancer samples (Cancer Genome Atlas Network, 2012) shows two mutational hotspots in PIK3CA within the accessory and catalytic kinase domains. (**B**) Output from waterfall shows mutations for five genes across 50 select TCGA breast cancer samples with mutation type indicated by colour in the grid and per sample/gene mutation rates indicated in the top and left sidebars. (**C**) Output from genCov displays coverage (bottom plots) showing focal deletions in sample A (last exon) and B (second intron) within a gene of interest. GC content (top plot) is encoded via a range of colours for each exon. (**D**) Output from lohSpec for HCC1395 (Griffith *et al.*, 2015), HCC38 and HCC1143 (Daemen *et al.*, 2013) breast cancer cell lines shows LOH events, across all chromosomes, shaded as dark blue. (**E**) Output from covBars shows cumulative coverage for 10 samples indicating that for each sample, at least ∼75% of targeted regions were covered at ≥ 35× depth. (**F**) Output from compIdent for the HCC1395 breast cancer cell line (tumor and normal) shows variant coverage (bottom plot) and SNP allele fraction (main plot) indicating highly related samples. Note that 4/24 positions are discrepant and likely result from extensive LOH in this cell line

The identification of small variants (SNVs and indels) within a genomic context is of paramount importance for the elucidation of the genetic basis of disease. Numerous tools and resources have been created to identify variants in sequencing data (Wang *et al.*, 2013). Conversely, few tools exist to visually display and summarize these variants across sample cohorts. Given a gene of interest, it is often useful to view variant occurrences in the context of the translated protein product (Zhang *et al.*, 2012). A variety of options exist to achieve this; however tools that offer both automation and flexibility to perform this task are lacking (Supplementary Table S1) (Griffith *et al.*, 2015; Leiserson *et al.*, 2015; Nilsen *et al.*, 2012; Yin *et al.*, 2012; Zhou *et al.*, 2015). The function lolliplot was developed to allow for precise control over visualization options (Fig. 1A). This includes the ability to choose Ensembl annotation databases for protein domain displays and to plot multiple tracks of mutations above and below the protein representation. Another common objective of genomic studies is to identify variant recurrence across multiple genes within a cohort. The GenVisR function waterfall was developed to calculate and rapidly illustrate the mutational burden of variants on both a gene and sample level, and further differentiates between variant types (Fig. 1B) (Krysiak *et al.*, 2016; Ma *et al.*, 2016; Wagner *et al.*, 2016). Mutually exclusive genomic events at the variant level are emphasized in this visualization by arranging samples in a hierarchical fashion such that samples with mutations in the most recurrently mutated genes are ranked first. Finally, it is often informative to investigate the rate of transition and transversion mutations observed across a set of cases. For example, lung tumors originating from patients with a history of tobacco smoke exposure display a pattern of enrichment for C to A or G to T transversions (Govindan *et al.*, 2012). The function TvTi (transversion/transition) was developed to improve recognition of these types of patterns within a cohort.

## 3 Visualization of copy number alterations

Copy number alterations occurring within the genome are implicated in a variety of diseases (Beroukhim *et al.*, 2010). The function GenCov illustrates amplifications and deletions across one or more samples in a genomic region of interest (Fig. 1C). A key feature of GenCov is the effective use of plot space, especially for large regions of interest, via the differential compression of various features (introns, exons, UTR) within the region of interest. For a broader view the function cnView plots copy number calls, and the corresponding ideogram, for an individual sample at the chromosome level. The function cnSpec displays amplifications and deletions on a still larger scale via copy number segments calls. This information is displayed as a heat map arranged in a grid indexed by chromosomes and samples. Alternatively, cnFreq displays the frequency of samples within a cohort that are observed to have copy number gains or losses at specific genomic loci. In addition to copy number changes, loss of heterozygosity (LOH) often plays an important role in genomic diseases. For example, in acute myeloid leukemia copy neutral LOH has been associated with shorter complete remission and worse overall survival (Gronseth *et al.*, 2015). The function lohSpec displays LOH regions observed within a cohort (Fig. 1D) by plotting a sliding window mean difference in variant allele fractions for tumor and normal germline variants.

## 4 Visualization of data quality

In genomic studies, the quality of sequencing data is of critical importance to the proper interpretation of observed variations. Therefore, we provide a suite of functions focused on data quality assessment and visualization. The first of these, covBars, provides a framework for displaying the sequencing coverage achieved for targeted bases in a study (Fig. 1E). A second function, compIdent, aids in the identification of mix-ups among samples that are thought to originate from the same individual (Fig. 1F). This is achieved by displaying the variant allele fraction of SNPs in relation to each sample. By default, 24 biallelic ‘identity SNPs’ (Pengelly *et al.*, 2013) are used to determine sample identity.

## 5 Example usage

GenVisR was developed with the naïve R user in mind. Functions are well documented and have reasonable defaults set for optional parameters. To illustrate, creating Figure 1B was as simple as executing the waterfall function call on a standard MAF (version 2.4) file containing variant mutation data and choosing which genes to plot:

genes = c(“PIK3CA”, “TP53”, “USH2”, “MLL3”, “BRCA1”)

GenVisR::waterfall(x=maf_file, plotGenes=genes)

The MAF file format originally developed for The Cancer Genome Atlas project (Cancer Genome Atlas Research Network, 2008) is the default file format accepted by waterfall. This format was chosen based on its simplicity and accessibility. A number of resources exist to convert VCF files common to most variant callers to MAF format. In the interest of maintaining flexibility, the waterfall and other GenVisR functions are able to accept alternative file types as input.

## 6 Conclusion

GenVisR provides features and functions for many popular genomic visualizations not otherwise available in a single convenient package (Table S1). By leveraging the abilities of ggplot2 (Wickham, 2009) it confers a level of customizability not previously possible. Virtually any aspect of a plot can be changed simply by adding an additional layer onto the graphical object. Thus, GenVisR allows for publication quality figures with a minimal amount of required input and data manipulation while maintaining a high degree of flexibility and customizability.

## Supplementary Material

Supplementary Data
