# Supplementary material for: GenVisR: Genomic Visualizations in R
Source: Bioinformatics. 2016 Jun 10;32(19):3012–4. doi: 10.1093/bioinformatics/btw325 (PMC5039916; doi:10.1093/bioinformatics/btw325)
Supplement: Supplementary Data [file supp_32_19_3012__index.html]

GenVisR: Genomic Visualizations in R — GenVisR: Genomic Visualizations in R — Supplementary Data 

# GenVisR: Genomic Visualizations in R

## Supplementary Data

files

- Supplementary Data - pdf file
