# Supplementary material for: GenVisR: Genomic Visualizations in R
Source: Bioinformatics. 2016 Jun 10;32(19):3012–4. doi: 10.1093/bioinformatics/btw325 (PMC5039916; doi:10.1093/bioinformatics/btw325)
Supplement: Supplementary Data [file supp_btw325_TableS1_software_comparison.pdf]

					Program					
**Feature Group**	**Feature**	**GenVisR**	**copynumber**	**GMS**	**ggbio**	**trackViewer**	**ProteinPaint**	**MutationMapper**	**OncoPrinter**	**MAGI**
**General**	**Language**	R	R	PERL/R	R	R	javascript	javascript	javascript	javascript
	**Allows customization of plot aesthetics**	Yes	Yes	No	Yes	Yes	Yes	No	No	Yes****
	**Visualization engine**	ggplot2	base R	base R/SVG	ggplot2	base R	D3	N/A	N/A	D3
	**Programatic access to functions**	Yes	Yes	No	Yes	Yes	No	No	Yes*	No
	**Simple installation/easy access to function**	Yes	Yes	No	Yes	Yes	Yes	Yes	Yes	Yes
	**Interactive**	No	No	No	No	No	Yes	Yes	Yes	Yes
	**Internet connectivity not required**	Yes**	Yes	Yes	Yes	Yes	No	No	No	No
	**Multiple Species support**	Yes	No	Yes	Yes	Yes	No	No	Yes	No
	**Plotted data exportable**	Yes	No	No	No	No	No	No	No	No
**Copy Number**	**Summarizes copy number frequency calls within a cohort**	Yes	Yes	No	No	No	No	No	No	No
	**Summarizes copy number calls within a cohort**	Yes	Yes	No	No	No	No	No	No	Yes
	**Plots copy number calls**	Yes	Yes	Yes	No	No	No	No	No	No
	**Plots copy number segments**	Yes	Yes	Yes	No	No	No	No	No	Yes
	**Supports genome wide copy number view**	Yes	Yes	Yes	No	No	No	No	No	No
	**Plots an ideogram**	Yes	Yes	Yes	Yes	No	No	No	No	No
	**Allows labeling of genes on copy number plot**	No	No	Yes	No	No	No	No	No	Yes
	**Calculates/displays Loss of Heterozygosity**	Yes	No	No	No	No	No	No	No	No
	**Displays coverage for a cohort in a region of interest**	Yes	No	No	Yes	No	No	No	No	No
	**Displays transcripts**	Yes	No	No	Yes	Yes	No	No	No	No
	**Displays GC content of transcript features**	Yes	No	No	No	No	No	No	No	No
	**Allows compression of transcript features**	Yes	No	No	Yes	No	No	No	No	No
	**Can reduce transcripts to a representative isoform**	Yes	No	No	Yes	No	No	No	No	No
	**Can display transcript strand**	No	No	No	Yes	Yes	No	No	No	No
**Data Quality**	**Efficiently display sequence coverage for genome**	Yes	No	Yes	Yes	No	No	No	No	No
	**Compares/displays sample relatedness**	Yes	No	No	No	No	No	No	No	No
**Small variants**	**Displays protein domains**	Yes	No	Yes	No	Yes***	Yes	Yes	No	Yes
	**Mutations next to each other are repulsed in protein display**	Yes	No	Yes	No	Yes	Yes	No	No	No
	**Allows for the display of variants on multiple tracks in protein display**	Yes	No	No	No	No	No	No	No	Yes
	**Allows control over variant display variables in protein display**	Yes	No	No	No	Yes	Yes	No	No	No
	**Displays Transition/Transversion frequency**	Yes	No	No	No	No	No	No	No	No
	**Displays clinical data in waterfall plot**	Yes	No	No	No	No	No	No	No	Yes
	**Displays mutation burden in waterfall plot**	Yes	No	No	No	No	No	No	No	No
	**Number of Mutation types allowed for display in waterfall plot**	No Limit	No	No	No	No	No	No	12	7
	**Flexible input format for waterfall plot**	Yes	No	No	No	No	No	No	No	Yes
	**Visualizes mutual exclusivity and co-occurrence between genes**	Yes	No	No	No	No	No	No	Yes	Yes
	**Allows for subsetting data within the function in waterfall plot**	Yes	No	No	No	No	No	No	No	No

*** Access is available via API**
**** Full functionality requires an internet connection for lolliplot**
***** Requires user to manually define domains**
****** Limited**

Good
Bad
Neutral

References

**Name**	**URL**	**Citation**
GenVisR	http://bioconductor.org/packages/GenVisR/	N/A
copynumber	http://bioconductor.org/packages/copynumber/	Nilsen, G., *et al.* Copynumber: Efficient algorithms for single- and multi-track copy number segmentation. *BMC Genomics* 2012;13:591.
GMS	https://github.com/genome/gms/	Griffith, M., *et al.* Genome Modeling System: A Knowledge Management Platform for Genomics. *PLoS Comput Biol* 2015;11(7):e1004274.
ggbio	http://bioconductor.org/packages/ggbio/	Yin, T., Cook, D. and Lawrence, M. ggbio: an R package for extending the grammar of graphics for genomic data. *Genome Biol* 2012;13(8):R77.
trackViewer	http://bioconductor.org/packages/trackviewer/	N/A
ProteinPaint	http://explore.pediatriccancergenomeproject.org/proteinPainter/	Zhou, X., *et al.* Exploring genomic alteration in pediatric cancer using ProteinPaint. *Nat Genet* 2015;48(1):4-6.
MutationMapper	http://www.cbioportal.org/mutation_mapper.jsp	N/A
OncoPrinter	http://www.cbioportal.org/oncoprinter.jsp	N/A
MAGI	http://magi.brown.edu/	Leiserson, M.D., *et al.* MAGI: visualization and collaborative annotation of genomic aberrations. *Nat Methods* 2015;12(6):483-484.
